# Prediction of Rice Plant Height Using Linear Regression Model by Pyramiding Plant Height-Related Alleles

**DOI:** 10.3390/ijms26136249

**Published:** 2025-06-28

**Authors:** Yongxiang Huang, Zhihao Xie, Daming Chen, Haomin Chen, Yuxiang Zeng, Shuangfeng Dai

**Affiliations:** 1 National Center for Technology Innovation of Saline-Alkali Tolerant Rice, Guangdong Ocean University, Zhanjiang 524008, China; hyx978025@gdou.edu.cn (Y.H.); yunsheng777@foxmail.com (Z.X.); 15976563623@stu.gdou.edu.cn (D.C.); chmstudy@foxmail.com (H.C.); 2 College of Coastal Agricultural Sciences, Guangdong Ocean University, Zhanjiang 524008, China; 3 State Key Laboratory of Rice Biology and Breeding, China National Rice Research Institute, Hangzhou 310006, China

**Keywords:** rice (*Oryza sativa* L.), plant height, association mapping, linear regression model

## Abstract

Although numerous rice plant height-related genes have been cloned and functionally characterized in recent years, a gap between the identified genes and their utilization in breeding still exists. Here, we developed a linear regression model by pyramiding plant height-related alleles to predict rice plant height and confirmed that it can be used in rice breeding. In our study, we firstly identified 22 plant height-associated molecular markers from 218 markers in an association mapping population which consisted of 273 rice varieties. Linear regression analysis revealed a positive correlation between rice plant height and the number of plant height-increasing alleles derived from these 22 molecular markers. Subsequently, linear regression models were developed using 2–10 loci based on the genotype and phenotype data of the association mapping population. The predictive accuracy of the model was tested using a recombinant inbred line (RIL) population consisting of 219 lines, and it revealed the trend that predictive accuracy increased with more loci in a certain range of less than five loci. If the prediction model was built based on 5–10 loci, it yielded an average absolute error from 11.05 to 11.96 cm, which was smaller than absolute error induced by environmental factors (5.72 cm to 12.79 cm). The reliable prediction of rice plant height by this model highlights its value as a practical tool for optimizing rice breeding strategies. Additionally, the linear regression model developed in this study not only can facilitate plant height manipulation but also will inspire other design breeding techniques in other crops or other traits.

## 1. Introduction

Plant height is an important trait which influences rice yield directly. Lodging usually causes yield loss which happens more frequently in extremely tall plants than it does in short plants. But short plants usually have lower yield than tall and strong plants. Therefore, there is a balance between plant height and rice yield. The high-yield rice varieties used in production have optimum plant height which can not only bear the weight of rice seed but also fight against lodging. Therefore, plant height is an important target of modern breeding [1].

Genetic studies have shown that rice plant height is controlled by genes with major effects or QTLs with minor effects [2,3]. More than 100 genes influencing rice plant height have been cloned and characterized in the past twenty years. These genes are involved in gibberellic acid biosynthesis [4,5,6,7,8]; catabolism, homeostasis, and the signal transduction pathway [9,10]; the brassinosteroid biosynthesis and signal transduction pathway; the strigolactone biosynthesis and signal transduction pathway [11,12,13,14]; the indole acetic acid pathway [15]; the auxin pathway [16,17]; the cytokinin pathway [18]; the ethylene biosynthesis pathways [19]; and other signal pathways [3]. Genes regulating rice plant height include transcription factor genes [2,4,6,20,21,22]; the E3 ubiquitin ligase-encoding gene [23,24]; the cytochrome P450 protein-encoding gene [25]; and so on. In addition, small RNA was also reported to be involved in the regulation of rice plant height [16,26].

Although great efforts have been made to uncover the plant height regulation mechanism in the past twenty years, manipulating these plant height-regulating genes in rice breeding is still a difficult task. Phenotype results from the interplay between genotype and the environment. Plant height, as a classic quantitative trait, is influenced by numerous genes with modest effects and is highly sensitive to environmental conditions [27]. However, pyramiding breeding, which means the stacking of beneficial alleles at multiple associated loci, can reduce environmental sensitivity and enhance the stability of the desired phenotypic outcome [28]. For example, the pyramiding of three high-cadmium-uptake loci in the rice variety TJN25-11 significantly enhanced cadmium phytoextraction efficiency [29], and pyramiding multiple genes of three key root trait could also aid breeding of flood-tolerant rice [30]. Pyramiding genes related to multiple excellent traits to achieve a higher level of comprehensive crop traits is the direction of future breeding [31]. However, from a breeding perspective, the ability to forecast phenotypic outcomes resulting from gene pyramiding would substantially improve its practical implementation and impact, especially for plant height, a key component of ideal plant architecture, influencing lodging resistance and yield potential. If we can fine-tune height by strategically combining genes from different loci, we may gain unprecedented precision in shaping this vital trait. Linear regression models enable the detection of coordinated multi-gene effects [32], and constructing linear regression models based on data on phenotype and genotype is a reliable method for accurately predicting phenotype after gene aggregation [33]. Zeng et al. developed a linear regression model using genotypic data from six loci and sheath blight resistance data from 273 rice varieties and demonstrated its predictive capability for lesion length in new rice populations based on genotype [33].

To facilitate the application of plant height-related genes in breeding programs, we first developed a linear regression model by pyramiding plant height-related alleles to predict rice plant height based on association mapping results of a mapping population which consisted of 273 rice genotypes. To test the prediction accuracy of the linear regression model, we used another recombinant inbred line (RIL) population, and the errors between the predicted value and the real plant height were close to the natural variation. It is suggested that this prediction model can be used in different breeding populations. In addition, we also tested the prediction accuracy of the model built by pyramiding alleles from two to ten loci, and there was a trend that using more loci yielded more accurate models. These results benefit the rice breeding community by providing an applicable rice plant height prediction strategy.

## 2. Results

### 2.1. Significant Loci Associated with Rice Plant Height Detected by Association Mapping

A mixed linear model (MLM) was employed to perform association analysis between 218 molecular markers and plant height traits across a mapping population of 273 rice varieties cultivated in five distinct environments (*p* < 0.01) (Table 1, Supplementary Figure S1, Supplementary Table S1). The analysis identified 35 significant marker-trait associations, involving 22 distinct molecular markers, with 8 markers showing consistent detection across multiple environments (Table 1, Supplementary Table S2). RM3589 and RM409 were repeatedly detected in four different environments; D1051 was repeatedly detected in three different environments; and five markers (D1126, D206C, D815, RM523, RM6103) were repeatedly detected in two different environments. Fourteen markers (D116C, D118A, D120A, D122E, D128A, D130B, D134B, D142A, D142C, D304B, D224C, D448, D622, D818) were detected only in one environment (Table 1).

### 2.2. Pyramiding Plant Height-Related Alleles Could Promote the Plant Height of Rice

**Figure 1:**
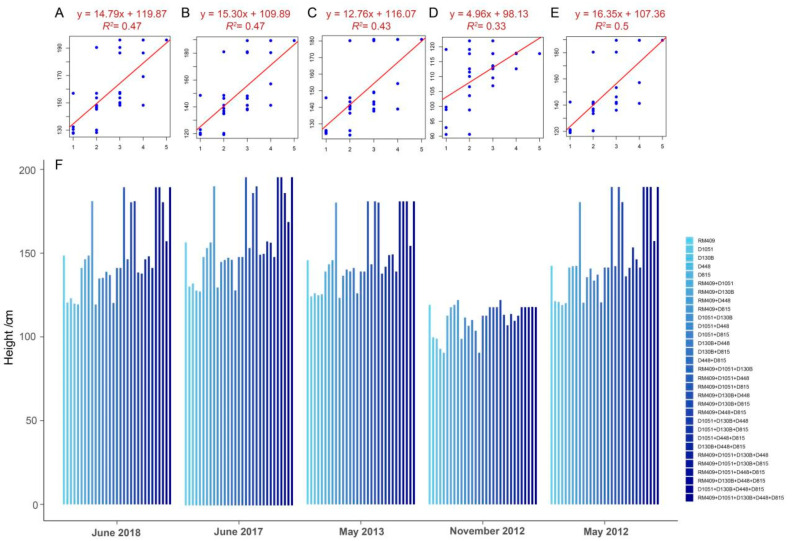
Correlation between plant height and the number of plant height-related loci (RM409, D1051, D130B, D448, and D815) in mapping populations planted in different years and locations. (**A**–**E**) represent the correlation between plant height and the number of loci when the 273 rice varieties were planted in different years and locations: (**A**) June 2018 in Hangzhou (F = 25.77; *p* = 0.0001); (**B**) June 2017 in Hangzhou (F = 25.87; *p* < 0.0001); (**C**) May 2013 in Hangzhou (F = 22.09; *p* < 0.0001); (**D**) November 2012 in Hainan (F = 14.40; *p* = 0.0007); (**E**) May 2012 in Hangzhou (F = 28.50; *p* < 0.0001). The x axis in Figure (**A**–**E**) indicates the number of pyramiding plant height-related alleles from one to five loci, and the y axis indicates the plant height (cm). (**F**) The average plant height of plants with different combinations of plant height-related loci in mapping population.

To further investigate whether pyramiding multiple favorable plant height-related loci had a promoting effect on plant height, five loci (RM409, D1051, D130B, D448, and D815) with relatively consistent effectiveness in different environments were selected and used to examine the correlation between plant height and the number of favorable loci (ranging from two to five) in the mapping population which consisted of 273 varieties (Supplementary Tables S2 and S3). The results showed that there was a trend of plant height increasing with more loci in different environments (Figure 1). This suggested that plant height can be manipulated by pyramiding plant height-related alleles at different loci.

### 2.3. Confirmation the Effect of the Five Plant Height-Related Loci in Influencing Plant Height by Using Another Recombinant Inbred Line (RIL) Population

**Figure 2:**
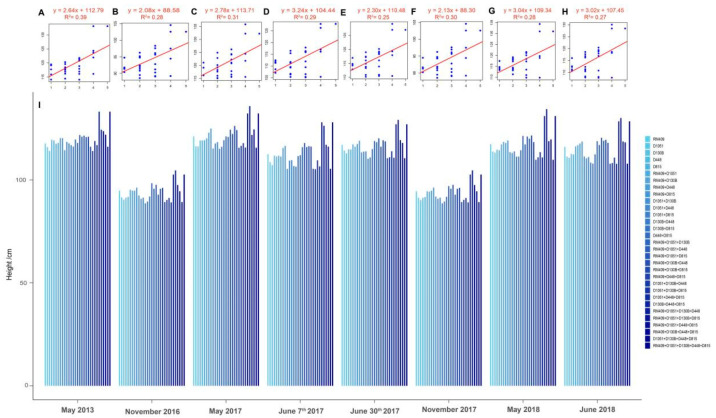
Lines with more plant height-increasing alleles pyramided at five loci (RM409, D1051, D130B, D448, and D815) had higher plant height in RIL population. (**A**–**H**) represent the regression analysis based on the number of plant height-related loci and plant height measured in eight different environments from 2013 to 2018, respectively: (**A**) May 2013 in Hangzhou (F = 18.53; *p* = 0.0002); (**B**) November 2016 in Hainan (F = 11.12; *p* = 0.0023); (**C**) 29 May 2017 in Hangzhou (F = 12.95; *p* = 0.0012); (**D**) 7 June 2017 in Hangzhou (F = 12.11; *p* = 0.0016); (**E**) 30 June 2017 in Hangzhou (F = 9.91; *p* = 0.0038); (**F**) November 2017 in Hainan (F = 12.23; *p* = 0.0015); (**G**) 23 May 2018 in Hangzhou (F = 11.14; *p* = 0.0023); (**H**) 2 June 2018 in Hangzhou (F = 10.63; *p* = 0.0028). The x axis indicates the number of plant height-increasing alleles from one to five loci in the RILs, and the y axis indicates the plant height (cm). (**I**) The average plant height (cm) of lines with different combinations of plant height-related loci in the RIL population.

The above analysis has demonstrated that five loci (RM409, D1051, D130B, D448, and D815) could be successfully used in marker-assisted breeding in the association mapping population which consisted of 273 genotypes (Figure 1). To estimate whether these five loci can be used for predicting plant height in other populations, another RIL population which consisted of 219 lines for further analysis was used (Supplementary Table S4). The RIL population was developed by crossing Lemont and Yangdao4, and their plant height traits were accurately obtained in eight different environments from 2013 to 2018 (Supplementary Table S5). The results of linear regression analysis on plant height and the number of plant height-related loci showed that lines carrying more plant height-increasing alleles at the five loci had higher plant height (Figure 2). It is suggested that these five loci can be used in marker-assisted breeding in other rice populations, which also testifies the potential of manipulating plant height by pyramiding plant height-related alleles at different loci.

### 2.4. Building Linear Regression Model for Prediction of Rice Plant Height Based on Plant Height-Related Alleles

The above research results show that plant height was influenced by the plant height-increasing alleles at five loci (Figure 1 and Figure 2). As is well known, plant height is a typical quantitative trait controlled by multiple loci with small effects (Table 1). If a linear regression model is developed for the prediction of rice plant height, how many plant height-related loci are required? To answer this question, we developed a prediction model using two to ten of the plant height-related marker loci and calculated the prediction accuracy of the model (Supplementary Table S2). We wanted to know whether the prediction model would be more accurate if more loci were used in building the model.

Plant height for a specific genotype varied when a plant was planted in different locations. Rice tended to have shorter plant height when planted in Hainan rather than in Hangzhou (Figure 1 and Figure 2). To minimize errors, we developed linear regression models using plant height data collected from the mapping population only grown in Hangzhou over four years (2012, 2013, 2017, and 2018) (Table 2). The genotypes of markers were transferred into the genotypic value at all the ten loci during building the linear regression models: the genotypic value 2 or −2 at one locus indicated that it contained plant height-increasing or -decreasing alleles, respectively (Supplementary Tables S3 and S5). The genotype and plant height data of the RIL population collected from six different conditions in Hangzhou were used to evaluate the prediction accuracy of the prediction model (Supplementary Table S5, Supplementary Figure S2). The average plant height of the RIL population grown over six environments in Hangzhou was 115.17 cm, which was considered the real plant height (Supplementary Table S5). We employed linear regression models incorporating varying numbers of loci to predict mean plant height in the RIL population, followed by validation against observed phenotypic values.

The results showed that if more than five loci were used in building the prediction model, the predicted average plant height values were already very close to real value, 115.17 cm (Table 2). We also compared the absolute errors between predicted plant height and the real plant height of each of the 219 RILs: the average absolute error of 219 lines reduced from 14.02 cm if the model was built with two loci to 12.82 cm if the model was built with three loci; and it reduced to 12.14 cm if the model was built with four loci and even reduced to 11.91 cm if the model was built with five loci (Table 2). The results showed a trend towards greater model accuracy with more loci used to construct the linear regression model in a certain range of less than five loci. Meanwhile, models incorporating more than five loci showed no significant improvement in prediction accuracy, because the average absolute errors between predicted plant height and the real plant height of the 219 RILs were all about 11.9 cm when the models were built using 5, 6, 8, and 10 loci, although the value decreased to 11.05 cm when using 9 loci, but the average absolute error of the 219 lines was 13.11 cm if 7 loci were used to build the prediction model (Table 2). Therefore, based on our comprehensive evaluation, a minimum of five genetic loci were necessary to develop a model with sufficient accuracy for rice plant height estimation.

### 2.5. Genotype-Environment Interaction Influence Rice Plant Height

Crop phenotypes arise from the complex interplay of genetic factors and environmental influences. Our initial analysis revealed a ~10% discrepancy between predicted and observed plant height values. To dissect whether this variation stems from model limitations or environmental effects, we leveraged multi-environment trial data (five distinct conditions) from the RIL population to quantify environmental contributions to plant height variation by using two-way analysis of variance (ANOVA) method. The results of two-way ANOVA showed that all three factors significantly contributed to plant height (*p* < 0.0001). Genotype contributed 76.44% to the total variance and the interaction between genotype and the environment contributed 19.95% of the total variance, while the environment alone only contributed 3.61% of the total variance (Table 3). This indicated that the interaction between genotype and the environment significantly contributed to plant height.

To further elucidate environmental effects on rice plant height, we conducted a temporal comparison of RILs cultivated in the same location across multiple growing seasons. The RIL populations used in our study were grown in six different years on the farm of China National Rice Research Institute in Fuyang, Hangzhou, and we found that the plant height of a specific line varied when grown in different years. We calculated the absolute differences in the plant height of these 219 lines grown between different years. It was found that the average absolute differences of the 219 lines grown between different years varied from 5.72 cm to 12.79 cm (Table 4). This suggested that absolute errors of 5.72 cm to 12.79 cm for the plant height of a rice genotype are normal and natural variance. Therefore, we believe that the linear regression model developed for plant height prediction, which had normal—about 11 cm—errors between the prediction value and the real value (Table 4), in our research was accurate for predicting rice plant height.

## 3. Discussion

Ideal plant architecture enhances photosynthetic efficiency, increases biomass production, and improves stress tolerance [34,35]. Plant height is one of the most important traits in rice ideal architecture. There is an optimum plant height for high-yield rice plants, because too much height would cause lodging and a height that is too short would result in low yielding [1,36]. If rice plant height could be predicted by using plant height associated loci, the precise regulation of rice plant height would be achieved in modern breeding process combined with gene editing or genes pyramiding breeding techniques.

As a classic quantitative trait, plant height is governed by numerous small-effect genes and exhibits significant environmental plasticity [8]. Linear regression modeling provides an effective approach to simultaneously evaluate the coordinated effects of these multiple genetic factors and generate predictive insights into their collective influence on trait variation [32,33]. In order to develop a molecular breeding technique to manipulate rice plant height, we firstly identified 22 molecular markers significantly associated with plant height among 218 markers in an association mapping population. The association mapping population contained 273 rice varieties from around the world and was grown in 5 different years for evaluation of plant height. The plant height was higher if an individual plant contained more plant height-increasing alleles at five plant height-related loci, whether in the association population consisting of 273 rice varieties or the RIL population consisting of 219 lines (Figure 1 and Figure 2), which is consistent with the concept of gene pyramiding breeding [37].

To determine the optimal number of loci for rice plant height prediction, we developed linear regression models incorporating 2–10 height-associated loci in the association mapping population and then validated these models by predicting plant height in the RIL population and comparing the predicted plant height with real observed measurements. The results showed a trend towards greater model accuracy with more loci used to construct the linear regression model in a certain range of less than five loci (Table 2). The average absolute differences in the plant height of the 219 RILs grown between different years varied from 5.72 cm to 12.79 cm, while the average absolute error between predicted plant height and the real plant height of the 219 lines was less than 11.96 cm if 5, 6, 8, 9, and 10 marker loci were used in building the linear regression model for plant height prediction, which suggested that the prediction errors were close to natural variance. Our analysis also revealed that incorporating 5 additional loci did not significantly improve model accuracy (Table 2), because the average absolute error between the predicted plant height and the actual plant height using the model constructed with 5, 6, 8, 9, and 10 loci was 11.91 cm, 11.91 cm, 11, 91 cm, 11.05 cm, and 11.96 cm, respectively (Table 2). Notably, the model constructed with seven markers performed worse (13 cm error) than models with fewer markers, producing errors larger than natural height variation (Table 2). This elevated error may have stemmed from the environmental susceptibility of the additional marker D448 incorporated in this model. Previous research has reported that a linear regression model construed with six rice sheath blight-related loci could accurately predict the sheath blight lesion length [33]; these results align with our findings that five or more trait-associated loci can yield models with sufficient predictive accuracy.

Plant height was significantly influenced by the genotype–environment interaction; the plant height of a specific genotype varied when the plant was grown in different years in the same location [3]. In our study, the RIL population was planted a total of six times in Hangzhou and two times in Hainan, and it is obvious to see that the plant height in plants from Hainan was significantly shorter than that in those from Hangzhou (Figure 2). Even when plants were only planted in Hangzhou, environmental factors induced significant plant height variation (5.72–12.79 cm) in genetically identical plants when grown in different years or at different locations within Hangzhou during the same year (Table 4), which fully demonstrated the characteristic of plant height as a quantitative trait [8]. Two-way ANOVA revealed that genotype accounted for 76.44% of plant height variance, the genotype–environment interaction for 19.95%, and the environment alone for only 3.61% (Table 3), which indicated that interaction between genotype and environment was the main cause of plant height variation. Previous studies emphasized the effect of the genotype–environment interaction as a quantitative trait [38,39]; our research on plant height also yielded similar results.

Although the prediction error of the linear regression model was very close to the natural plant height variance, we believe that there are still some ways to improve this model. Here are some strategies: (1) We supposed that all the alleles at different plant height-related loci had equal contributions to the plant height phenotype. But different alleles at different loci should have different influences on plant height. We suggest using different weighting for different alleles to refine the prediction model in future analysis. (2) We suggest choosing the best marker loci for building the prediction model, also paying attention to the epistatic interaction at different loci. In our results, we found that epistatic interaction existed between the RM409 locus and the D815 locus, because plants reached the greatest height (180 to 190 cm) if they carried plant height-increasing alleles at both loci (Table 1, Figure 1). So we must pay attention to epistasis when building the prediction model. In addition, we believe that there exist the most suitable marker loci for building the prediction model. For instance, choosing seven loci did not yield a good prediction result compared to choosing only three loci (Table 2). Finding the best loci for building the prediction model is a tough job if calculating by hand. We suggest developing a computer program to find the most suitable loci with the best prediction accuracy. (3) The prediction model was built based on the plant height phenotypic data attained in the same location, in Hangzhou. Therefore, the prediction model can only used to predict the plant height phenotype of plants grown in Hangzhou. We do not know whether it can be used to predict the plant height of rice populations grown in other locations. For example, if we want to predict the height of rice plants grown in Beijing, the original plant height phenotypes attained in Beijing must be used to build the prediction model first. This is the limitation of the prediction model and it needs to be improved in the future to be applicable in more environments.

In summary, we identified 22 plant height-associated markers from a set of 218 molecular markers and developed linear regression models capable of predicting plant height in a training population (the mapping population consisted of 273 varieties). The model demonstrated strong predictive accuracy when applied to an independent test population (the RIL population consisted of 219 lines). This approach aligns with the core principles of genome-wide selection (GS) breeding, serving as a proof-of-concept for implementing GS-based breeding strategies [40,41]. Our results suggest that with sufficient genetic marker data, this method can enable precise trait prediction in crops, thereby enhancing breeding efficiency and significantly accelerating varietal development.

## 4. Materials and Methods

### 4.1. Association Mapping Population for Detecting Plant Height-Related Marker Loci and Building Plant Height Prediction Model

The association mapping population consisted of 273 rice genotypes (Supplementary Tables S1 and S3). The names and detailed information of the 273 genotypes have been described by Zeng et al. [39]. Briefly, the 273 genotypes originated from 15 counties and consisted of landraces, cultivars, breeding lines, deep-water rice, upland rice, weedy rice, *Oryza sativa*/*O. rufipogon* descendants, japonica, indica, and javanica rice and represented the diversity of rice genetic variation (Supplementary Figure S1). The 273 varieties were planted in four different years (May 2012, May 2013, June 2017, and June 2018) at the farm of China National Rice Research Institute (CNRRI) in Fuyang district, Hangzhou (119°95′ E, 30°07′ N), and they were planted in November 2012 at the trial station of CNRRI in Lingshui, Hainan (110°02′ E, 18°48′ N), for the phenotyping of rice plant height. At least 18 individual plants were planted for each rice genotype in different growing environments. The 18 individuals were grown in one plot for each genotype, the plot locations were completely randomized [39]. Three individual plants were randomly selected in each plot for plant height phenotyping in a mature stage.

### 4.2. Recombinant Inbred Line Population for Evaluating Plant Height Prediction Model

A recombinant inbred line (RIL) population which consisted of 219 lines was developed by crossing Lemont, an American japonica variety, with Yangdao 4, a Chinese indica variety, and it was used to evaluate the plant height prediction model. This RIL population was planted in six environments on the CNRRI farm in Fuyang district, Hangzhou (119°95′ E, 30°07′ N), and two environments in the CNRRI trial station in Lingshui, Hainan (110°02′ E, 18°48′ N), for the phenotyping of rice plant height. The six environments in Hangzhou included (1) F6 in May 2013, (2) F13 on 29 May 2017, (3) F13 on 7 June 2017, (4) F13 on 30 June 2017, (5) F15 on 23 May 2018, and (6) F15 on 2 June 2018. The two planting environments in Hainan included (1) F12 in November 2016 and (2) F14 in November 2017. The plant height phenotyping of the RIL population was evaluated in the same way as the association mapping population mentioned above was.

### 4.3. Association Mapping Using TASSEL

A total of 218 markers, which consisted of 7 SSR markers and 211 Indel markers, were used to assay the 273 rice genotypes. The primer sequences and more detailed information of the 218 markers have been described by Zeng et al. [33]. The rice leaf DNA extraction and PCR protocols followed those of Zeng et al. [42]. The PCR products were separated on 8% denaturing polyacrylamide gels as described by Zeng et al. [33] and visualized by silver staining [43]. Standard marker bands were added to each gel as controls to compare the band size of the PCR product, and the Image Lab software Version 5.2.1 build 11 (Bio-Rad Laboratories, Hercules, CA, USA) was also used to determine the size of the PCR products [33].

Structure (v2.3.1, Stanford University, Stanford, CA, USA) was used to calculate the population structure and the Q matrices [44] based on the marker genotype (Supplementary Tables S6 and S7). The Q matrices were used for association mapping with the mixed linear model in TASSEL (v3.0.174, Cornell University, Ithaca, NY, USA) [45]. Significant marker–trait association was determined by a mixed linear model with a *p* value lower than 0.01 [33].

### 4.4. Building Linear Regression Model for Prediction of Plant Height

A linear regression model was used to predict plant height:

y = ax + b

where y is the predicted value of plant height, x is sum of the genotypic values of different plant height-related loci, a is the regression coefficient, and b is the intercept.

The genotypic value of each plant height-related loci were set to be 2 or −2: 2 indicated that a locus contained homozygous plant height-increasing alleles, while −2 indicated that it contained homozygous plant height-decreasing alleles. We used the genotypic values of the plant height-related loci and the phenotypic data of an association mapping population which consisted of 273 rice genotypes to build the linear regression model for the prediction of plant height. The linear regression model was built by using SAS software (v8.01, University of California, Berkeley, CA, USA) [46].

### 4.5. Evaluation of the Prediction Accuracy of the Prediction Model

Since the plant height prediction model was built by using an association mapping population, it had to be tested in another different population for examining the potential of this model in production. We used an RIL population which contained 219 lines to test the plant height prediction model. The predicted plant height of each of the 219 lines was compared with the real plant height data, and the average absolute error of all the 219 lines was calculated:

$$\mathrm{AAE}=\frac{\sum_{i=1}^{n}\left|\left(P-R\right)\right|}{n}$$

where AAE is the average absolute errors of the linear regression model, P is the predicted plant height of a specific line in the RIL population, R is the real plant height of this line, and n = 219 which indicates 219 lines.

### 4.6. Influence of Interaction Between Genotype and Environment on Plant Height Phenotype

Two-way ANOVA was calculated using SAS (v8.01, University of California, Berkeley, CA, USA) [46] to test whether the interaction between genotype and the environment had a significant effect on plant height. The contribution of genotype, the environment, and the genotype–environment interaction to the total plant height variances were also calculated. The average absolute variance of the plant height of the 219 RILs grown between different years was calculated to examine the variance of plant height in different years.

## 5. Conclusions

We developed a linear regression model to predict rice plant height by using the genotypic and phenotypic data of an association mapping population which consisted of 273 rice genotypes. This prediction model was tested in a recombinant inbred line (RIL) population with 219 lines to examine its prediction accuracy. The average absolute error between the prediction values and real plant height was 11.96 cm (smaller than the natural plant height variance of the RIL population) if the model was built by using 5, 6, 8, 9, and 10 loci. We believe that this prediction model had great potential in rice breeding. The prediction model was more accurate if more loci were used.

## Figures and Tables

**Table 1:** Significant marker–trait associations detected in five different environments.

Planting Time and Location	Marker	Chr.	F Value	*p* Value	Marker R^2^	Allele Associated with Highest Plant Height (Marker Band Size in bp)
May 2012 in Hangzhou	D815	8	10.72	0.0013	0.059	130
May 2012 in Hangzhou	D1051	10	10.58	0.0014	0.058	96
May 2012 in Hangzhou	RM409	9	5.99	0.0030	0.064	95
May 2012 in Hangzhou	D224C	2	7.50	0.0068	0.040	88
May 2012 in Hangzhou	D206C	2	5.07	0.0072	0.054	175
May 2012 in Hangzhou	D1126	11	7.28	0.0076	0.039	175
May 2012 in Hangzhou	D818	8	4.75	0.0097	0.052	180
November 2012 in Hainan	D142C	1	6.36	0.0004	0.094	225
November 2012 in Hainan	D448	4	11.80	0.0007	0.058	140
November 2012 in Hainan	RM3589	7	4.91	0.0008	0.096	100
November 2012 in Hainan	RM523	3	4.43	0.0019	0.091	152
November 2012 in Hainan	D118A	1	6.12	0.0026	0.060	150
November 2012 in Hainan	D116C	1	5.38	0.0053	0.053	165
November 2012 in Hainan	D622	6	7.82	0.0057	0.038	135
November 2012 in Hainan	D120A	1	5.24	0.0060	0.051	178
November 2012 in Hainan	D134B	1	5.00	0.0076	0.049	115
November 2012 in Hainan	D128A	1	4.96	0.0079	0.049	100
November 2012 in Hainan	D122E	1	4.83	0.0089	0.047	112
November 2012 in Hainan	D142A	1	4.80	0.0092	0.047	203
November 2012 in Hainan	D1051	10	6.86	0.0095	0.037	96
May 2013 in Hangzhou	RM3589	7	4.95	0.0008	0.096	100
May 2013 in Hangzhou	RM523	3	3.83	0.0050	0.076	152
May 2013 in Hangzhou	RM409	9	5.37	0.0054	0.052	95
June 2017 in Hangzhou	D1051	10	10.78	0.0012	0.044	96
June 2017 in Hangzhou	RM3589	7	4.65	0.0012	0.072	100
June 2017 in Hangzhou	RM409	9	6.85	0.0013	0.053	95
June 2017 in Hangzhou	D1126	11	7.40	0.0070	0.028	175
June 2017 in Hangzhou	RM6103	3	7.14	0.0080	0.027	188
June 2018 in Hangzhou	RM409	9	11.10	0.0000	0.083	95
June 2018 in Hangzhou	RM3589	7	5.32	0.0004	0.080	100
June 2018 in Hangzhou	RM6103	3	9.88	0.0019	0.037	188
June 2018 in Hangzhou	D130B	1	5.76	0.0036	0.043	105
June 2018 in Hangzhou	D304B	3	4.43	0.0047	0.050	180
June 2018 in Hangzhou	D815	8	7.82	0.0055	0.030	130
June 2018 in Hangzhou	D206C	2	4.79	0.0091	0.037	175

**Table 2:** Prediction of the RIL population’s plant height using linear regression models constructed by using the association mapping population.

Number of Loci Used in Building the Prediction Model	F Value (*p* < 0.0001)	Plant Height Prediction Model Developed Using the 273 Rice Genotype *	Predicted Average Plant Height of the 219 RILs (cm)	Average Plant Height of the 219 RILs Grown in Six Years (cm)	Average Absolute Error Between Predicted Plant Height and the Real Plant Height of the 219 RILs (cm)
2 (RM409, RM6103)	42.23	y = 5.47816 x + 137.12733	123.85	115.17	14.02
3 (RM409, RM6103, D130B)	30.09	y = 2.60955 x + 129.0469	121.85	115.17	12.82
4 (RM409, RM6103, D130B, D224C)	24.06	y = 1.59275 x + 126.14526	120.89	115.17	12.14
5 (RM409, RM6103, D130B, D224C, D142C)	39.89	y = 1.92841 x + 127.87717	117.66	115.17	11.91
6 (RM409, RM6103, D130B, D224C, D142C, RM3589)	47.99	y = 1.97065 x + 131.53479	117.15	115.17	11.91
7 (RM409, RM6103, D130B, D224C, D142C, RM3589, D448)	77.65	y = 2.45987 x + 131.22406	113.63	115.17	13.11
8 (RM409, RM6103, D130B, D224C, D142C, RM3589, D448, D1051)	57.79	y = 1.74358 x + 128.60196	115.07	115.17	11.91
9 (RM409, RM6103, D130B, D224C, D142C, RM3589, D448, D1051, D1126)	31.42	y = 1.14012 x + 126.32044	116.50	115.17	11.05
10 (RM409, RM6103, D130B, D224C, D142C, RM3589, D448, D1051,D1126, D815)	52.69	y = 1.54178 x + 126.80416	116.60	115.17	11.96

* x and y in the prediction model indicate genotypic value and plant height, respectively.

**Table 3:** Two-way analysis of variance of plant height in 219 genotypes of an RIL population grown in five different environments in Hangzhou.

	DF	Type 1 SS	Mean Square	F Value	*p*	% of Genotype + Environment + Genotype × Environment
Genotype	218	531,630.10	2438.67	227.09	<0.0001	76.44
Environment	4	25,125.86	6281.47	584.94	<0.0001	3.61
Genotype × environment	872	138,735.51	159.10	14.82	<0.0001	19.95

**Table 4:** The average absolute differences (cm) in plant height of 219 lines grown between different years in the same location Hangzhou.

	Planted in May 2013	Planted on 29 May 2017	Planted on 7 June 2017	Planted on 30 June 2017	Planted on 23 May 2018	Planted on 2 June 2018
Planted in May 2013	-	12.56	12.08	11.53	11.82	12.79
Planted on 29 May 2017		-	11.11	9.25	9.79	10.67
Planted on 7 June 2017			-	8.33	8.37	7.25
Planted on 30 June 2017				-	6.96	7.95
Planted on 23 May 2018					-	5.72
Planted on 2 June 2018						-

## Data Availability

Data are available from the corresponding author upon request.

## Supplementary Materials

The following supporting information can be downloaded at https://www.mdpi.com/article/10.3390/ijms26136249/s1.
