# Supplementary material for: Prediction of Rice Plant Height Using Linear Regression Model by Pyramiding Plant Height-Related Alleles
Source: Int J Mol Sci. 2025 Jun 28;26(13):6249. doi: 10.3390/ijms26136249 (PMC12249533; doi:10.3390/ijms26136249)
Supplement: Supplementary file 1 [file ijms-26-06249-s001.zip › ijms-3688625-Supplementary figures.pdf]

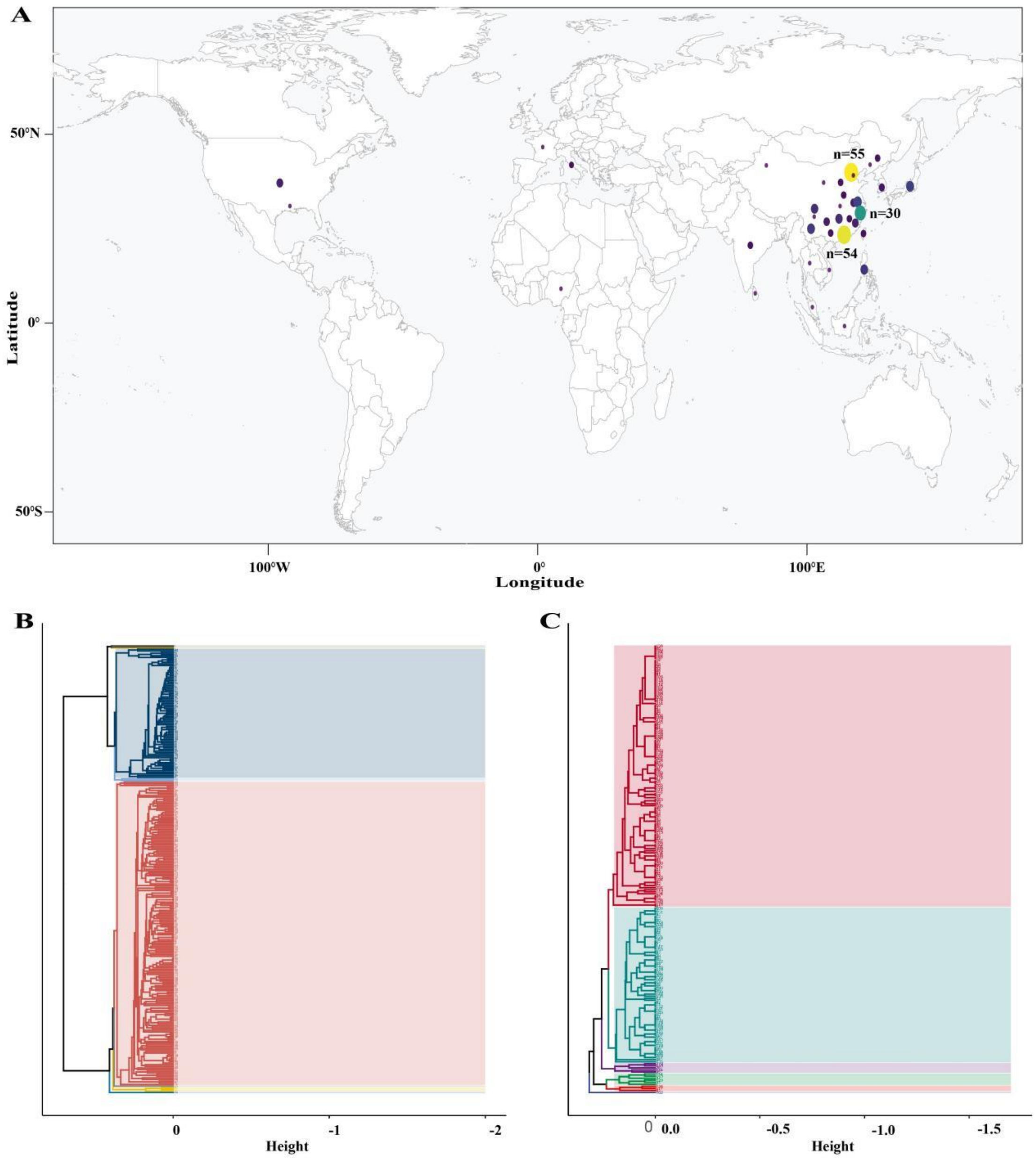

Supplementary Figure S1. The distribution and cluster analysis of the materials used in this study. (A) The distribution of the mapping population consisting of 273 varieties from around the world. The size of the points in the figure reflects the sample size. Points with a sample size greater than 30 are marked with different colors and the lowercase letter "n" in the figure represents the number of samples. (B) The UPGMA clustering of 273 varieties based on 218 markers. (C) The UPGMA clustering of 219 RIL lines based on 10 markers.

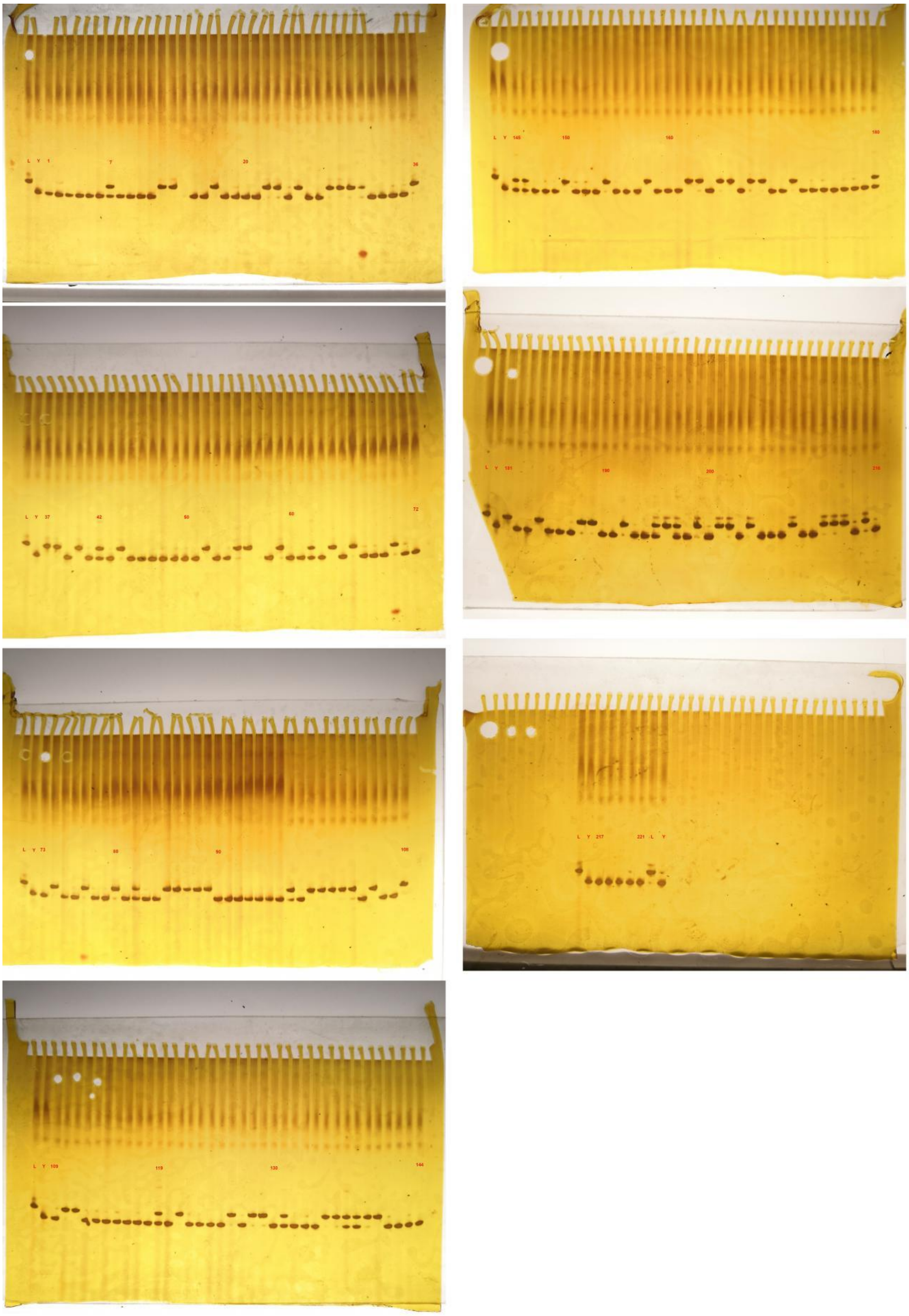

Supplementary Figure S2. Electropherograms from the polyacrylamide gel electrophoresis (PAGE) of the marker D224C in 219 RIL population lines. L denotes Lemont, the maternal parent of the RIL population. Y denotes Yangdao4, the paternal parent of the RIL population. Numbers above the bands indicate RIL numbers. For example, "1" corresponds to the "1" line in Supplementary Tables S4 and S5.
